# Intestinal Fibrosis in Crohn’s Disease: Pathophysiology, Diagnosis, and New Therapeutic Targets

**DOI:** 10.3390/jcm14124060

**Published:** 2025-06-08

**Authors:** Fotios S. Fousekis, Konstantinos Mpakogiannis, Ioanna Nefeli Mastorogianni, Georgios D. Lianos, Dimitrios K. Christodoulou, Konstantinos H. Katsanos

**Affiliations:** 1 Department of Gastroenterology and Hepatology, University Hospital of Ioannina, University of Ioannina, 45110 Ioannina, Greece; kostismpakogiannis@gmail.com (K.M.); nefelimastorogianni@gmail.com (I.N.M.); dchristodoulou@gmail.com (D.K.C.); khkostas@hotmail.com (K.H.K.); 2 Department of Surgery, University Hospital of Ioannina, 45110 Ioannina, Greece; georgiolianos@yahoo.gr

**Keywords:** Crohn’s disease, intestinal fibrosis, extracellular matrix, anti-fibrotic therapy

## Abstract

Crohn’s disease (CD) is a chronic inflammatory disorder of the gastrointestinal tract that often leads to intestinal fibrosis, an irreversible complication associated with strictures and the need for surgical intervention. Fibrosis occurs due to prolonged inflammation and abnormal wound healing, involving complex interactions between immune cells, mesenchymal cells, cytokines, and the gut microbiota. Key fibrogenic mechanisms include the activation of fibroblasts and myofibroblasts, cytokine signaling, and disrupted turnover of the extracellular matrix. Advancements in imaging techniques, such as MRI and CT enterography, have improved the detection and monitoring of fibrosis. Additionally, molecular techniques targeting fibroblast activation proteins show promise as a new imaging method. However, there are currently no approved anti-fibrotic therapies for CD. Emerging strategies focus on key pathways and novel therapeutic targets, including growth factor modulators, intracellular enzyme and kinases modulators, and interventions targeting the modulation of inflammation and extracellular matrix, which are being evaluated in preclinical and clinical settings. This review discusses the pathophysiology, diagnostic advancements, and therapeutic perspectives related to intestinal fibrosis in CD, emphasizing the urgent need for targeted anti-fibrotic therapies to prevent long-term complications and improve the life quality of patients.

## 1. Introduction

Crohn’s disease (CD) is a chronic inflammatory disorder of the gastrointestinal tract characterized by a relapsing and remitting course, affecting patients’ quality of life. Fibrotic strictures represent a significant complication of CD, manifesting in approximately 50% of patients within a five-year period following diagnosis, and in approximately 70% of patients within a ten-year period [1]. Intestinal fibrosis is characterized by excessive accumulation of extracellular matrix components, arising from prolonged intestinal inflammation and impaired tissue repair. This process leads to stiffening and/or scarring of the affected tissue, resulting in obstructive symptoms in patients with CD [2,3]. The strictures are usually located in the ileum and the ileocolonic region, which may be attributed to the comparatively reduced diameter of the ileum in comparison to the colon. Nevertheless, strictures may develop at any site affected by CD, including the upper gastrointestinal tract, the colon and rectum. The location and frequency of strictures is likely to reflect the distribution of inflammation [4]. In contrast to inflammation, which can be managed with medical treatments, such as immunosuppressants and biologics, intestinal fibrosis is frequently irreversible and presents a significant therapeutic challenge. The annual incidence of hospitalizations of patients with CD appears to be 20%. Furthermore, half of these patients require surgery intervention within 10 years after diagnosis. Postoperative recurrence has been observed in 44–55% of cases after 10 years [5]. A large portion of these surgical interventions are attributed to stricturing disease. This underscores the need for a holistic approach to understanding and treating intestinal fibrosis in CD.

The pathophysiology underlying fibrostenotic CD is multifactorial and involves a complex interplay between immune cells, mesenchymal cells, and gut microbiota [6,7]. Chronic intestinal inflammation triggers a signaling cascade that activates fibroblasts and myofibroblasts, promoting the excess deposition of collagen and other extracellular matrix components. In particular, growth factors such as transforming growth factor-beta (TGF-β) and connective tissue growth factor (CTGF) appear to be central to the drive of these fibrotic processes by enhancing fibroblast proliferation and collagen synthesis and accumulation [8,9]. Over time, persistent intestinal inflammation, combined with repeated cycles of tissue damage and repair, creates an environment that promotes irreversible structural changes [10].

In this review, we aim to explore the key mechanisms underlying intestinal fibrosis in CD, focusing on the cells and molecular pathways involved in the pathogenesis of fibrosis. In addition, we present the diagnostic approaches for fibrotic CD. Furthermore, we discuss and analyze the potential of novel anti-fibrotic therapies.

## 2. Literature Research

A comprehensive literature search was conducted using the PubMed and MEDLINE databases to provide an overview of this field, focusing on articles published in English up to February 2025. The keywords and search phrases included “treatment AND fibrosis AND Crohn’s disease”, “diagnosis AND intestinal fibrosis”, “pathogenesis AND intestinal fibrosis AND Crohn’s disease”, “Mechanisms of fibrosis AND Crohn’s disease”. This strategy provided a comprehensive selection of studies focused on the pathophysiological mechanisms, diagnostic advancements, and potential therapeutic targets for intestinal fibrosis in CD. The search also aimed to uncover new insights into the cellular and molecular pathways that contribute to fibrotic progression, as well as to identify emerging therapeutic strategies.

## 3. Mechanisms of Fibrosis in Crohn’s Disease

**Figure 1:**
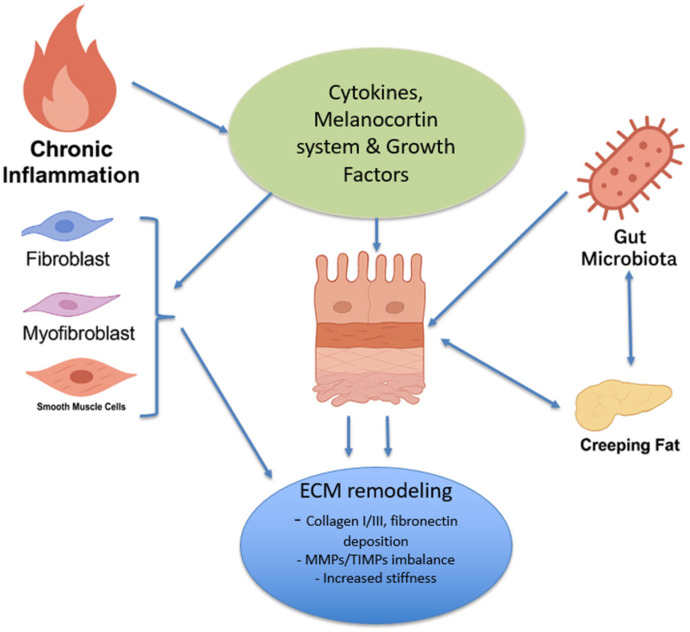
The schematic representation of the key pathogenic mechanisms driving intestinal fibrosis in Crohn’s disease. Chronic inflammation, dysregulation of cytokines, growth factors, and the melanocortin system, along with fibroblast, myofibroblast, and smooth muscle cell activation, contribute to excessive extracellular matrix (ECM) remodeling. Additionally, alterations in the gut microbiota and creeping fat hypertrophy amplify immune responses and fibrotic progression, leading to sustained tissue damage and architectural disruption.

Intestinal fibrosis in Crohn’s disease is a multifaceted process, involving a complex interplay of various factors (Figure 1) (Table 1). Understanding the mechanisms behind fibrosis is critical for developing targeted therapies.

### 3.1. Cells Involved in Intestinal Fibrosis

Intestinal fibrosis in CD arises from the complex interaction of various cells and molecular pathways. Key players such as fibroblasts, myofibroblasts, and macrophages, and signaling molecules, such as TGF-β, drive tissue remodeling. Mesenchymal cells, including fibroblasts, myofibroblasts, and smooth muscle cells, play a key role in intestinal fibrosis. Their proliferation and activation are triggered by bioactive factors like growth factors and cytokines [31]. Fibroblasts are essential for maintaining epithelial stem cells, regulating immune homeostasis and supporting endothelial functions [32]. Three main fibroblast subsets appear to regulate homeostasis by producing key molecules. CD81+ fibroblasts maintain intestinal stem cell identity via WNT ligands, R-spondins, and Gremlin 1. PDGFRαhi fibroblasts promote epithelial differentiation via BMPs and WNT5A, while PDGFRαloCD81- fibroblasts contribute to extracellular matrix production and remodeling [33]. Fibroblasts isolated from IBD mucosa were found to proliferate faster than fibroblasts from the control intestine. Collagen secretion from IBD fibroblasts, regardless of type, was shown to be increased compared to control fibroblasts and PDGF, and bFGF and TGF-beta1 induced collagen secretion from IBD fibroblasts [11]. Additionally, activated fibroblasts transform into myofibroblasts that express α-SMA as they migrate along the fibrin lattice into the wound [34].

Myofibroblasts play a central role in intestinal fibrosis and originate from various cellular origins. These include bone fibrocytes, marrow-derived mesenchymal cells, pericytes, and epithelial and endothelial cells. Additionally, they can arise from transitions such as epithelial to mesenchymal transition and endothelial to mesenchymal transition [12,35,36]. Myofibroblasts are identified by specific intracellular proteins, including α-smooth muscle actin (α-SMA), type 3 intermediate filaments like vimentin or desmin, and collagen type 1 maturation enzymes, while lacking epithelial cytokeratins [37]. Myofibroblasts from stenotic bowel have been shown to differ in both phenotype and function from those from normal or inflamed bowel of the same patient with CD. Stenotic myofibroblasts show higher expression of genes related to extracellular matrix modulation and collagen deposition. In a fibrotic environment, normal myofibroblasts increase matrix metalloproteinases (MMPs) expression to counteract matrix forces, whereas stenotic myofibroblasts paradoxically decrease MMP3 expression [13].

Intestinal smooth muscle cells appear to be actively involved in the formation of intestinal fibrosis. Smooth muscle cells primarily come from resident mesenchymal cells. However, they can also originate from differentiated endothelial cells via endothelial-to-mesenchymal transition and from differentiated epithelial cells through epithelial-to-mesenchymal transition. Additional sources include intestinal stellate cells and bone marrow-derived stem cells and fibrocytes [38,39]. These cells can be identified through positive staining for vimentin (low), α-SMA, desmin, and collagen type I [2,40]. Chronic inflammation promotes their activation, leading to increased production of extracellular matrix, growth factors, and cytokines. These factors not only drive tissue remodeling but also stimulate the proliferation of surrounding cells [14,15]. Specifically, cytokines from smooth muscle cells enhance their own proliferation, contributing to hyperplasia and hypertrophy. There is bidirectional signaling between SMCs and myofibroblasts that is influenced by the microenvironment. Both cell types secrete factors that influence each other’s activation and proliferation [14,39].

### 3.2. Molecular Mechanisms in Intestinal Fibrosis

In order to understand the progression of intestinal fibrosis, it is essential to explore the main molecular mechanisms involved. In the following section, we will analyze the roles of specific cytokines and growth factors that orchestrate this pathological process.

Cytokines are small, soluble proteins secreted by cells such as lymphocytes, natural killer cells, and macrophages. Cytokines are essential mediators in the immune response, regulating the growth and activity of immune cells. A single cytokine can be produced by different cell types and can affect multiple cell types, resulting in various biological effects [41]. Certain cytokines have been found to play a crucial role in the development of fibrosis in CD. Key pro-fibrotic mediators include IL-1, IL-6, IL-13, IL-4, and IL-17, which promote fibroblast activation, epithelial–mesenchymal transition, and collagen deposition [16]. IL-13 plays a particularly central role by signaling through IL-13Rα and inducing the production of TGF-β and collagen production [42]. Additionally, IL-21 and IL-33 enhance fibrotic responses [43]. On the other hand, certain cytokines like IL-7, IL-10, and IL-22 appear to have anti-fibrotic effects. They function by modulating TGF-β signaling and inhibiting collagen synthesis [17,44,45]. The IL-23/IL-17 axis appears to play an important role in intestinal inflammation and homeostasis [2,46]. However, its role in inflammation-induced fibrosis is not fully clarified. Evidence indicates that members of the IL-17 cytokine family may contribute to intestinal fibrosis. IL-17A is upregulated in strictured areas of the gut in CD patients [47] and may directly interact with IL-17 receptor-expressing myofibroblasts, playing a significant role in stricture development [48]. Overall, the progression and severity of fibrosis depend on the balance between pro-fibrotic and anti-fibrotic cytokines [39].

Transforming growth factor-β (TGF-β) plays a crucial role in cellular responses, including immunity, differentiation, and proliferation. TGF-β appears to be the primary regulator driving fibrosis in many organs, including the intestine [8,34]. TGF-β binds to betaglycan (type III receptor), forming a complex with the type II receptor, which then activates the type I receptor. This triggers phosphorylation of Smad2/3, which forms a complex with Smad4. The Smad complex moves to the nucleus to regulate TGF-β target genes, including collagen type I and fibronectin [49]. TGF-β has been identified to be expressed in the gastrointestinal tract, including epithelial cells and mesenchymal cells [50]. TGF-β and its receptors are elevated in the intestinal cells of patients with IBD, especially in those with CD. In mice, overexpression of TGF-β leads to colonic fibrosis [51]. The overexpression of TGF-β2 may promote fibrosis by enhancing extracellular matrix deposition through local myofibroblasts and fibroblasts that are derived from fibroblasts [8]. In a mouse model, a TGF-beta1 peptide-based vaccine that inhibited excessive TGF-beta1 activity was found to prevent intestinal fibrosis [52].

Platelet-derived growth factor (PDGF), produced by various cell types, including smooth muscle cells, endothelial cells, and fibroblasts, has been linked to fibrotic disorders [53]. Elevated PDGF levels have been detected in the inflamed mucosa of IBD, especially in CD and collagenous colitis [54]. In the colonic mucosa, PDGF-B stimulates intestinal myofibroblast migration and proliferation [18]. In addition, increased PDGF-B levels have been detected in the serum of IBD patients, promoting the proliferation of human colon fibroblasts in vitro. Biopsies from CD patients also show significant upregulation of PDGF receptor-beta (PDGFR-β), with positive staining for PDGF ligands and receptors near ulcerations [53].

Connective tissue growth factor (CTGF) is a cysteine-rich peptide secreted by fibroblasts when activated by transforming growth factor beta (TGF-beta). CTGF acts as a downstream mediator of TGF-beta’s effects on connective tissue cells, stimulating the synthesis of extracellular matrix and cell proliferation [19].

Fibroblast growth factors (FGFs) are a group of polypeptides essential for embryonic development and postnatal functions, including injury response, regulation of cell excitability, and metabolism. Depending on the subfamily, FGFs can function in different ways: intracellularly, paracrinely, or endocrinely [55]. Basic FGF (bFGF) is a potent mitogen involved in wound healing and fibrosis, especially in CD, where higher levels of bFGF are associated with the thickening of the intestinal wall, suggesting a possible involvement of bFGF in the process of transmural fibrogenesis in CD [56].

The melanocortin system is a network of molecular mediators and receptors that regulate various physiological processes, including melanogenesis, steroidogenesis, neuromodulation, and inflammation. It operates through five melanocortin receptors (MC1-5R), which are G protein-coupled receptors [57,58]. Melanocortins directly regulate inflammation by inhibiting the NF-κB family of proteins, which control genes involved in producing cytokines like TNF, as well as related receptors, adhesion molecules, and chemokines [57]. Recent studies indicate that melanocortins contribute to inflammation mechanisms in IBD and tissue damage, which leads to intestinal fibrosis development. Melanocortin 3 and 5 receptors (MC3R and MC5R) are expressed at higher levels in the inflamed mucosa of CD compared to normal mucosa [59]. Furthermore, evidence suggests that melanocortin receptors (MCRs) are present in various types of fibroblasts, actively participating in the endogenous regulation of their functions [20].

### 3.3. Alterations of Extracellular Matrix

The extracellular matrix is a dynamic structure found in all tissues, undergoing controlled remodeling to maintain tissue architecture and homeostasis. Its main components include collagen proteins, non-collagen proteins, proteoglycans, growth factors, and enzymes. The extracellular matrix acts as a framework for cells and plays a crucial role in processes such as migration, cellular proliferation, and adhesion [60,61]. Additionally, extracellular matrix acts as a reservoir for signaling molecules involved in the fibrotic response to tissue injury. This includes macromolecules like fibronectin and collagens, as well as proteases, growth factors, and cytokines. Increased tissue stiffness prompts activated cells to deposit more extracellular matrix in fibrotic lesions [31]. In CD, chronic inflammation may disrupt the balance between extracellular matrix synthesis and degradation, leading to uncontrolled fibrotic tissue accumulation. Specifically, disturbed extracellular matrix remodeling during CD is associated with an increased activity of MMPs. MMPs participate in processes such as angiogenesis and the migration of inflammatory cells. The elevated activity of MMPs leads to extracellular matrix degradation and increased release of its components into the bloodstream [62]. In fibrotic intestinal tissue, increased levels of MMP-1, -3, and -14 are detected, primarily in the submucosal and muscular layers, along with elevated levels of the tissue inhibitor of MMPs-1 (TIMP-1), suggesting a possible counterbalance of extracellular matrix compartment degradation [21,22]. Notably, although MMP levels appear to be elevated in patients with fibrotic CD, the overall balance between MMPs and their inhibitors (TIMPs) is shifted towards the inhibition of extracellular matrix degradation [23].

Another component of the extracellular matrix that is altered in CD is hyaluronan. The synthesis of hyaluronan is increased during inflammation. However, in chronic conditions such as IBD, high-molecular-weight hyaluronan is degraded into smaller fragments that may promote inflammatory responses, inhibit fibroblast differentiation, and promote angiogenesis [61,63]. High-molecular-weight hyaluronan is a crucial component of the glycocalyx that excludes other molecules and cells, acting as an anti-angiogenic factor [64]. It interacts with surface receptors to prevent immune recognition and block phagocytosis by macrophages. In healthy tissues, high-molecular-weight hyaluronan maintains tissue integrity and suppresses inflammation [65]. However, in conditions like IBD, it breaks down into smaller fragments that can activate macrophages and dendritic cells, promoting inflammation by enhancing the expression of genes like IL-1β, IL-12, and TNF-a [66]. Evidence indicates that hyaluronan, in addition to regulating angiogenesis and inflammation, is crucial for the development of fibrous tissue. It appears to be a key contributor to TGF-β-induced scar formation [67,68].

### 3.4. Creeping Fat

Creeping fat, a distinct feature of CD, is characterized by the hypertrophy of mesenteric adipose tissue that wraps around the intestine, sometimes covering up to >50% of the bowel’s circumference [69]. Creeping fat is formed as a consequence of mesenteric adipose tissue responding to the production of inflammatory mediators and bacterial invasion through the intestinal mucosa [70]. This tissue appears to play an active role in fibrogenetic and inflammatory processes. In particular, creeping fat contains various cell types, including immune cells, adipocytes, and adipocyte progenitors [69]. These cells produce cytokines, such as IL-6, TGF-β, and IFN-γ, which drive excess extracellular matrix deposition and promote intestinal fibrogenesis [71]. Studies have shown that in CD, TGF-β levels are significantly increased in mesenteric adipose tissue, with activation of the Smad2/3 signaling pathway, leading to excessive extracellular matrix synthesis in mesenteric adipose tissue [72,73]. Furthermore, the interplay between creeping fat and the gut microbiota may also contribute to intestinal fibrosis in CD. The transmural inflammation of CD allows for an interaction between mesenteric fat and translocated intestinal microorganisms, thus contributing to the activation of the immune response [24,25]. This process results in the activation of pattern recognition receptors (PRRs) in adipocytes and resident immune cells, initiating an immune response. As a result, adipocytes hypertrophy and secrete pro-inflammatory cytokines such as IL-6 and TNF-α, as well as chemokines that recruit immune cells, including macrophages and T cells [24,26]. These immune cells and adipocytes maintain inflammation and fibrosis by producing large amounts of pro-fibrotic cytokines, adipokines, growth factors, and fatty acids [74]. Thus, creeping fat in CD may be a dynamic contributor to intestinal fibrosis through its secretion of cytokines, extracellular matrix remodeling, immune cell recruitment, and interaction with the gut microbiota.

### 3.5. Gut Microbiota and Intestinal Fibrosis

Postoperative fecal diversion procedures, such as colostomy or ileostomy, may be useful in alleviating clinical symptoms by resolving perianal disease and promoting mucosal healing [75,76,77]. Furthermore, antibiotic treatment has been demonstrated to improve the clinical outcomes of CD, indicating that eliminating certain bacterial populations positively affects the CD course [6,78,79]. Genetic research has identified variations in the NOD2 gene as a significant risk factor for CD, particularly in cases that affect the ileum [80]. NOD2 functions as an intracellular pattern recognition receptor that detects bacterial components and activates the NF-κB pathway, initiating immune responses [27].

Recent evidence suggests that persistent colonization by adherent-invasive *Escherichia coli* (AIEC) plays a significant role in intestinal fibrosis, a serious complication of CD. AIEC takes advantage of inflammation to maintain colonization, promoting fibrosis through the IL-33-ST2 signaling pathway [81]. Furthermore, the translocation of pathogen-associated molecular patterns (PAMPs) through the gut epithelium activates subepithelial myofibroblasts, leading to NF-κB-driven cytokine and chemokine secretion. This interaction, mediated by Toll-like receptors (TLRs) and NOD2, enhances fibroblast contraction and proliferation, potentially driving intestinal stricture formation [82]. Specifically, activation of TLR4 (responsive to Gram-negative bacteria) and TLR5 (recognizing flagellin) promotes a pro-fibrotic phenotype in intestinal fibroblasts, further exacerbating tissue remodeling in CD [28,82,83]. A study in pediatric patients with CD found that the presence of anti-I2 (directed against Pseudomonas fluorescens), anti-outer membrane protein C (directed against outer membrane porin C of *E. coli*), anti-CBir1 flagellin (an antibody directed against bacterial flagellin, a component of the gut microbiota), and anti-Saccharomyces cerevisiae antibodies were associated with a higher risk of stricturing disease [84]. Another study demonstrated that anti-CBir1 antibody reactivity was independently associated with fibrostenotic CD [85].

Growing evidence highlights the role of gut microbiota metabolites in the development of intestinal fibrosis in CD. The gut microbiota produces a variety of metabolites, including short-chain fatty acids (SCFAs), bile acids, and tryptophan derivatives [86]. These metabolites appear to play a crucial role in regulating immune responses, maintaining epithelial barrier integrity, and influencing fibrotic processes [87]. Alterations in microbial composition may result in modifications in bacterial metabolome, contributing to intestinal fibrosis. SCFAs, particularly butyrate, acetate, and propionate, are generated through the bacterial fermentation of dietary fiber. These metabolites may enhance the intestinal barrier, reduce inflammation, and provide energy to epithelial cells [29,30]. As a consequence of the dysbiosis found in CD, there is a decrease in SCFA-producing bacteria, such as Faecalibacterium prausnitzii [88,89]. In an animal model, extracellular vesicles derived from Faecalibacterium prausnitzii alleviate intestinal fibrosis related to chronic colitis by reprogramming macrophage metabolism [90].

## 4. Diagnosis of Intestinal Fibrosis

Timely and accurate diagnosis of intestinal fibrosis in CD is crucial for disease management and the prevention of severe complications, such as stricture formation and the need for surgical interventions. Due to the lack of ideal non-invasive diagnostic tools, endoscopy with biopsies remains the gold standard method while also identifying neoplastic lesions and providing an evaluation of CD activity and assessment of treatment efficacy [91].

In recent years, imaging techniques have been developed that may be used to identify intestinal fibrosis in patients with CD (Table 2). Advances in magnetic resonance imaging (MRI) have led to significant improvements in the detection and characterization of fibrosis. Techniques, such as native T1 mapping and magnetization transfer imaging, appear to be a promising imaging biomarker in grading the intestinal fibrosis of CD [92]. Additionally, the diffuse weighted imaging sequence with apparent diffusion coefficients and diffusion kurtosis imaging may be valuable in identifying fibrosis in the bowel of CD patients, enhancing diagnostic accuracy [93,94].

Ultrasound has been demonstrated to be a valuable diagnostic tool in detecting strictures and the application of novel techniques such as contrast-enhanced ultrasound and elastography are promising methods to differentiate between fibrotic and inflammatory strictures [95,96]. However, they present limitations for use in routine clinical practice. Ultrasound is both operator- and patient-dependent, and certain bowel segments, such as the proximal ileum and jejunum pelvic loops, are difficult to scan due to intestinal gas, common in chronic stricture patients. Furthermore, evaluating long or multiple strictures presents challenges, particularly with quantitative assessments requiring repeated measurements and clarity on which stenotic segment to assess [97].

Computed Tomographic Enterography (CTE) has shown promise in identifying intestinal fibrosis in patients with Crohn’s disease. Recent advancements, such as the combination of unenhanced multi-parametric spectral CT and 3D-printing techniques, appear to be effective in evaluating the extent and severity of fibrotic changes [98]. Additionally, emerging machine learning-based radiomic and deep machine learning model approaches applied to CTE have demonstrated improved predictive performance for intestinal fibrosis compared to traditional assessments by radiologists. This offers improved diagnostic accuracy [99,100].

Recent advances in molecular imaging have identified fibroblast activation protein (FAP) as a promising target for visualizing fibrotic tissue. FAP plays a key role in tissue remodeling and is often upregulated in fibrotic lesions, making it an appropriate biomarker for imaging fibrosis in CD [101]. Gallium-68 labeled FAP inhibitors (Ga-68-FAPI) used in PET/CT imaging appear to be a promising imaging method to differentiate inflammation from fibrosis and to guide subsequent therapy in patients with stricturing Crohn’s disease; however, further studies are warranted [102,103].

## 5. Anti-Fibrotic Treatment and Novel Targets

Despite the development of new biologics and small molecules in recent years, the treatment of fibrostenotic CD remains a challenge. Medical therapy is considered first-line treatment for inflammatory stenotic CD, but is ineffective for fibrostenotic CD. This is one of the main reasons that a high proportion of CD require surgery. In cases of symptomatic fibrotic strictures, an endoscopic or surgical approach should be considered. The choice of method should be based on the length, severity, and location of the stricture. Both endoscopic stricturoplasty and balloon dilation are minimally safe and minimally invasive procedures, with only a low risk of complications [104]. Surgical intervention is recommended for patients who are not candidates for endoscopic therapy to address fibrostenotic disease. This may involve a resection of the stenotic segment or strictureplasty [105]. Additionally, a third surgical option, bypass surgery, is primarily indicated for strictures located in the gastroduodenal region [106].

As our understanding of the pathophysiology of intestinal fibrosis continues to improve, new research into effective therapies is emerging. Currently, there is no therapy to treat or reverse intestinal fibrosis in CD; however, several preclinical studies have shown promising results [107]. The most promising anti-fibrotic targets and the specific molecules under investigation are highlighted in the following section.

### 5.1. Growth Factor Modulators

#### 5.1.1. TGF-β Inhibitors

TGF-β1 may play a crucial role in pathophysiological pathway of intestinal fibrosis through the activation of fibroblasts and an increase in extracellular matrix production. Pirfenidone, a downregulator of TGF-β in gene expression, has demonstrated inhibiting fibrosis, having already been approved for idiopathic pulmonary fibrosis [108]. In vitro data has demonstrated that pirfenidone may inhibit the growth of intestinal fibroblasts and it reduces collagen I production through the TGF-β1/mTOR/p70S6K signaling pathway, potentially offering a novel and safe approach for treating intestinal fibrosis [109]. In a murine colitis model, the administration of pirfenidone reduced collagen deposition in colitis-associated fibrosis by inhibiting the proliferation of colonic fibroblasts and TGF-β signaling pathways [110]. Other TGF modulators, such as avotermin and PRM-151, have demonstrated anti-fibrotic activity in various organs, including the skin and lungs [111,112]. However, there is currently insufficient data on their effects against intestinal fibrosis.

#### 5.1.2. Connective Tissue Growth Factor Inhibitors

Connective tissue growth factor (CTGF), also known as cellular communication network factor 2 (CCN2), appears to play a crucial role in the development and progression of fibrosis. Increased levels of CTGF have been observed in patients suffering from fibrotic diseases [113]. Furthermore, the elevated expression of CTGF in fibroblasts from strictured CD patients highlights its significant role in pathogenesis of intestinal fibrosis [9]. Notably, CTGF expression significantly increases not only at the site of inflammation but also in the distended segment proximal to the inflamed area [114]. Pamrevlumab (FG-3019) is a human monoclonal antibody targeting CTGF and has been approved for the treatment of idiopathic pulmonary fibrosis [115]. Despite advancements in our understanding of CTGF’s role in fibrogenesis and the development of targeted therapies like pamrevlumab, there is still a significant lack of clinical evidence assessing their effectiveness in treating intestinal fibrosis associated with Crohn’s disease.

#### 5.1.3. Fibroblast Growth Factor Analogues

Fibroblast growth factor analogues such as NGM282 (FGF19) and pegbelfermin (FGF21) have demonstrated efficacy in reducing hepatic fibrosis and inflammation [116,117]. Fibroblast growth factors are involved in regulating extracellular matrix dynamics in the intestine, highlighting the potential for their use in treating Crohn’s disease-related fibrosis [118].

### 5.2. Intracellular Enzymes and Kinases

Intracellular kinases and enzymes appear to play a crucial role in regulating various cellular functions and are implicated in the development of inflammatory and fibrotic conditions [119,120]. Recent studies using animal models have identified new molecular targets that may have therapeutic potential against intestinal fibrosis. These targets include mitogen-activated protein kinase (MAPK) pathways, Rho-associated protein kinases (ROCKs), endothelin-1 inhibitors, and AMPK/mTOR pathway.

MAPK signaling pathways play a crucial role in regulating various cellular functions and are involved in the development of inflammatory bowel disease. Apoptosis signal-regulating kinase 1 (ASK1) is a type of MAPK. In a mouse model of NBS-induced colitis, ASK1 has been shown to control the development of intestinal inflammation by regulating innate immunity [121]. Additionally, a randomized phase 2 trial demonstrated improvements in inflammation and fibrosis in patients with nonalcoholic steatohepatitis [122].

Rho kinases (ROCKs) are important in TGFβ-induced myofibroblast activation and represent potential therapeutic targets. In a mouse model, local ROCK inhibition has been demonstrated to prevent and reverse fibrosis by reducing myocardin-related transcription factor and p38 mitogen-activated protein kinase activation while enhancing autophagy in fibroblasts [123]. Similarly, targeting phosphatidylinositol 3-kinase (PI3K) p110δ activity in SHIP-deficient mice reduced intestinal fibrosis, including muscle thickening, mesenchymal cell accumulation, and collagen deposition. These findings suggest that PI3K p110δ inhibition could be an effective therapeutic strategy for intestinal fibrosis [124].

Endothelin-1 (ET-1) is a powerful endogenous vasoconstrictor primarily produced by endothelial cells [125]. Endothelins may play a role in the development of Crohn’s disease, as high levels of endothelin-1 have been observed in patients with CD and UC compared to healthy controls [126]. The use of bosentan, a drug that blocks endothelin receptors, has been shown to reduce the severity of intestinal inflammation induced by iodoacetamide and to enhance the healing of inflamed intestinal tissue [127]. While the anti-fibrotic effects of endothelin receptor antagonists have been demonstrated in various extra-intestinal target organs such as the heart and lungs [128], evidence on their action in the gut remains scarce and further studies need to be conducted.

The AMPK/mTOR pathway regulates autophagy and is crucial for maintaining intestinal integrity. Activating AMPK signaling may inhibit mTOR phosphorylation, enhance autophagy, and reduce intestinal mucosal damage. This pathway is an important therapeutic target for intestinal inflammation and is also linked to regulating fibrotic diseases. Modifying the AMPK/mTOR pathway and activating autophagy may help reduce intestinal inflammation and fibrosis [129]. In a TNBS-induced colitis model, total flavone of Abelmoschus manihot inhibited migration, proliferation, and collagen synthesis in intestinal fibroblasts. Moreover, it enhanced autophagy and apoptosis of intestinal fibroblasts through upregulated AMPK expression and decreased mTOR levels [130].

### 5.3. Inflammation Modulators

TNF-a is a cytokine, which plays a key role in the inflammation cascade of Crohn’s disease. Anti-TNF agents have revolutionized the treatment of IBD and are used to both induce and maintain remission in patients with CD. While their role in managing intestinal fibrosis seems to be limited, there is some evidence suggesting that these agents may contribute to the treatment of patients with intestinal fibrosis [131,132]. It has been found that TNF-a may promote collagen accumulation and proliferation in intestinal myofibroblasts through TNF receptor 2 [133]. Furthermore, anti-TNFα has been shown to prevent bowel wall inflammation and fibrosis in the peptidoglycan–polysaccharide rat model of Crohn’s disease, assessed through messenger RNA, histology, and magnetization transfer MRI [134]. A multicenter retrospective study found that anti-TNF agents are effective in about 25% of patients with CD and symptomatic intestinal strictures [135]. Another multicenter study demonstrated that a significant response to adalimumab was observed in approximately two-thirds of CD patients with symptomatic small bowel stricture and over half of the patients remained surgery-free four years after starting treatment [136]. However, it remains unclear whether the clinical response observed in patients with stenotic disease treated with anti-TNFs is attributable to a reduction in the inflammatory component of the stenoses or to regression of the underlying intestinal disease. Further studies are required to elucidate this distinction.

IL-36 is part of the IL-1 superfamily, consisting of three agonists (IL-36α, IL-36β, IL-36γ) and one antagonist (IL-36Ra). The agonists act as pro-inflammatory cytokines, promoting immune cell infiltration and the secretion of inflammatory molecules through the IL-36 receptor (IL-36R) [137]. Moreover, the data indicates a molecular pathway that involves the activation of IL36R in gut resident fibroblasts and the expression of MMP13 in the development of intestinal fibrosis [138]. In addition, higher levels of IL36A have been found in fibrotic intestinal tissues of patients with inflammatory bowel disease (IBD) when compared to control individuals. IL36 induces the expression of genes that regulate fibrogenesis in fibroblasts. Blocking or knocking out the IL36R gene in mice alleviates chronic colitis and intestinal fibrosis, suggesting that IL36R inhibitors could be potential treatments for intestinal fibrosis in IBD patients [139].

Regarding the remaining interleukins, several play important roles in the inflammation cascade in IBD; however, there is limited evidence supporting a potential anti-fibrosis role of interleukin modulators and further studies are required. IL-13 is a key driver of fibrosis through IL-13 receptors and TGF-β1 signaling, with increased expression observed in fibrotic intestinal tissue [42,140]; however, anti-IL-13 therapies have demonstrated limited clinical efficacy [141,142]. IL-10 has been demonstrated to reduce inflammatory cytokines and TGF-β1 in the IL-10 knockout model of CD, showing a promising trend in reducing tissue fibrosis [44], though no clinical trials have been conducted in fibrostenotic CD patients. IL-1α promotes fibrotic pathways by inducing TGF-β1, whereas IL-1β has variable effects on collagen production, suggesting potential novel targets against intestinal fibrosis [143,144].

Tumor necrosis factor-like cytokine 1A (TL1A), a member of the TNF superfamily, appears to be a crucial mediator in intestinal inflammation and fibrogenesis [145]. Its interaction with death receptor 3 (DR3) seems to drive fibrotic responses [146,147]. Transgenic mouse models that continuously express TL1A exhibit increased collagen deposition and fibroblast activation in the gut, particularly in the ileum and colon [148]. These effects are further intensified in the presence of gut microbiota, indicating a complex interaction between the host and microbes in the development of fibrosis [149]. Notably, neutralizing TL1A with monoclonal antibodies has been shown to reverse established fibrosis in animal studies, underscoring its potential as a therapeutic target [150]. Recent research shows that TL1A enhances the function of fibroblasts through the Rho/ROCK signaling pathway and promotes epithelial–mesenchymal transition, which is a critical factor in tissue remodeling [151]. In patients with IBD, elevated levels of TL1A are associated with increased mesenchymal markers and a loss of epithelial integrity [152]. Together, these findings suggest that targeting TL1A-DR3 signaling could not only help manage inflammation but also prevent or reverse fibrosis in CD. This positions TL1A as a promising candidate for future anti-fibrotic treatments in the context of inflammatory bowel disease [145].

### 5.4. Extracellular Matrix Modulators

Intestinal fibrosis results mainly from an imbalance in the deposition and degradation of extracellular matrix, which is regulated by MMPs and TIMPs. Targeting the regulation of these mechanisms may help to develop new drugs and achieve therapeutic goals. Fibroblast activation protein (FAP) is involved in fibrosis by regulating extracellular matrix deposition. Treatment with an anti-FAP antibody led to a dose-dependent decrease in type I collagen and TIMP-1 in stenotic tissues, while not affecting MMP-3 and MMP-12 secretion in strictures from individuals with CD when tested ex vivo [153]. Stenotic myofibroblasts exhibited decreased MMP3 expression and increased levels of the collagen cross-linking enzyme lysyl oxidase (LOX), leading to greater extracellular matrix contraction compared to normal and inflamed myofibroblasts [154]. Notably, in vivo LOX inhibition restored MMP3 activity and prevented excessive extracellular matrix contraction in a fibrotic environment. These findings suggest LOX inhibition as a potential anti-fibrotic treatment [13].

## 6. Conclusions

Intestinal fibrosis is a significant and irreversible complication of CD, contributing to morbidity and the need for surgical intervention. In contrast to inflammation, there are no effective medical treatments for fibrosis, highlighting the urgency of targeted anti-fibrotic therapies. The pathogenesis of intestinal fibrosis is multifactorial, involving a complex interplay of immune responses, mesenchymal cell activation, gut microbiota, and extracellular matrix remodeling. Cytokines, as well as growth factors such as CTGF and PDGF, drive fibroblast proliferation and collagen deposition. Advances in imaging techniques, including MRI and molecular imaging targeting fibroblast activation protein, offer potential for non-invasive diagnosis and monitoring of fibrotic progression. In recent years, novel therapeutic approaches targeting specific fibrogenic pathways are under investigation. A deeper understanding of the mechanisms driving intestinal fibrosis will open the way for the development of new drugs to halt or reverse fibrotic progression in CD.

## Figures and Tables

**Table 1:** Key pathogenic mechanisms and contributors to intestinal fibrosis in Crohn’s disease.

Component	Biological Mediators and Features	Pathogenic Role	Ref.
**Cellular Components**	- Fibroblasts, myofibroblasts, smooth muscle cells - Activation via TGF-β, PDGF, /EndoMT	Produce ECM; sustain inflammation and promote fibrosis	[11,12,13,14,15]
**Cytokines, Melanocortin System and Growth Factors**	- Pro-fibrotic: IL-13, IL-17, IL-1, TGF-β, CTGF, PDGF - Anti-fibrotic: IL-10, IL-22	Regulate fibroblast activation, collagen synthesis, and ECM turnover	[16,17,18,19,20]
**ECM Remodeling**	- Collagen I/III, fibronectin deposition - MMPs/TIMPs imbalance - Increased stiffness	Alters tissue architecture; favors chronic fibrotic progression	[21,22,23]
**Creeping Fat**	- Mesenteric fat hypertrophy - Secretion of IL-6, TNF-α, TGF-β - Interaction with microbiota	Amplifies inflammation; promotes ECM production and immune cell recruitment	[24,25,26]
**Gut Microbiota**	- AIEC colonization - NOD2, TLR4/5 activation - SCFA depletion (e.g., butyrate)	Triggers innate immune pathways and myofibroblast activation	[27,28,29,30]

AIEC: adherent-invasive Escherichia coli; CTGF: connective tissue growth factor; ECM: extracellular matrix; EndoMT: endothelial to mesenchymal transition; MMPs: matrix metalloproteinases; PDGF: platelet-derived growth factor; SCFA: short-chain fatty acids; TIMPs: tissue inhibitor of MMPs.

**Table 2:** Diagnostic methods for intestinal fibrosis in Crohn’s disease.

Diagnostic Method	Advantages	Limitations
**Endoscopy with biopsy**	Gold standard; allows histological analysis, detection of neoplastic lesions, and assessment of CD activity	Expensive; not always widely available; advanced sequences may require expertise
**Magnetic resonance imaging (MRI)**	High-resolution imaging; native T1 mapping and magnetization transfer imaging improve fibrosis grading	Expensive; not always widely available; advanced sequences may require expertise
**Ultrasound (contrast-enhanced, elastography)**	Non-invasive and accessible; can differentiate inflammatory vs. fibrotic strictures	Operator- and patient-dependent; limited by bowel gas and anatomic constraints
**Computed Tomographic Enterography (CTE)**	Enhanced by spectral CT and 3D printing; radiomic and machine learning analysis outperforms radiologist assessment	Radiation exposure; needs standardization for fibrosis-specific interpretation
**Molecular imaging (FAPI-PET/CT)**	Targets FAP expression; may distinguish fibrosis from inflammation with high specificity	Still experimental; requires further clinical validation
